# Nothing else matters: Video games create sustained attentional selection away from task-irrelevant features

**DOI:** 10.3758/s13414-020-02122-y

**Published:** 2020-09-11

**Authors:** Joe Cutting, Paul Cairns, Gustav Kuhn

**Affiliations:** 1 Digital Creativity Labs, University of York, York, UK; 2 Department of Computer Science, University of York, York, UK; 3 Department of Psychology, Goldsmiths, University of London, London, UK

**Keywords:** Attention: Selective, Attention: Divided Attention and Inattention, Attention: Interactions with Memory

## Abstract

Feature-based attention allocates resources to particular stimulus features and reduces processing and retention of unattended features. We performed four experiments using self-paced video games to investigate whether sustained attentional selection of features could be created without a distractor task requiring continuous processing. Experiments 1 and 2 compared two versions of the game *Two Dots*, each containing a sequence of images. For the more immersive game post-game recognition of images was very low, but for the less immersive game it was significantly higher. Experiments 3 and 4 found that post-game image recognition was very low if the images were irrelevant to the game task but significantly higher if the images were relevant to the task. We conclude that games create sustained attentional selection away from task-irrelevant features, even if they are in full view, which leads to reduced retention. This reduced retention is due to differences in attentional set rather than a response to limited processing resources. The consistency of this attentional selection is moderated by the level of immersion in the game. We also discuss possible attentional mechanisms for the changes in recognition rates and the implications for applications such as serious games.

## Introduction

Attention allows us to selectively process information by diverting cognitive resources towards the attended stimulus and away from other unattended stimuli (Carrasco, 2011; Desimone & Duncan, 1995). As such, attention plays a pivotal role in our conscious perception, and understanding its cognitive mechanisms has important theoretical and practical implications. Much of the research on attention has examined participants’ ability to process stimuli outside of their current attentional selection (e.g., Lavie et al., 2014; Mack & Rock, 1998; Simons & Chabris, 1999). Our attentional system operates on multiple levels of information processing streams. At its earliest level, our attentional selection is driven by what our eyes fixate on (i.e., overt attention), but looking at an object does not necessarily imply you will perceive it. Research on inattentional blindness (IB) has shown that if covert attention is sufficiently engaged on the main task then participants may not consciously perceive the presence of irrelevant stimuli even when this occurs at fixation (Mack & Rock, 1998; Most et al., 2001; Simons & Chabris, 1999).

Attention can also be selected on or away from particular stimulus features such as color, orientation or movement, which is known as *feature-based* attentional selection (Carrasco, 2011; McAdams & Maunsell, 2000). Feature-based selection impacts visual search (Carrasco et al., 1998) and perceptual performance (Liu et al., 2007). Feature-based selection has also been found to impact task-irrelevant processing within an inattentional blindness paradigm (Most & Astur, 2007; Most et al., 2001; Simons & Chabris, 1999). Most (2010) considered that both change blindness and inattentional blindness are aspects of similar phenomena in which stimuli are not perceived or remembered due to differences in attentional selection. He proposed dividing them into two types; *spatial* IB in which attention is diverted from particular spatial areas, and *central* IB in which attention is diverted from particular features of the stimulus. Wolfe (1999) has argued that inattentional blindness could be due to attentional moderation of memory rather than perception, and so should be seen as “inattentional amnesia.” Butler and Klein (2009) found evidence that some IB affects are due to attentional moderation of memory, but most investigations (e.g., Kuhn & Findlay, 2010; Rees et al., 1999; Ward & Scholl, 2015) have found that IB is due to attentional moderation of perception rather than memory.

Investigating task-irrelevant processing requires a method of keeping participants’ attention directed on the relevant task and ensuring that other stimuli are unattended. In many inattentional blindness experiments (e.g., Mack & Rock, 1998) participants were required to direct their attention on an attentionally demanding task (such as judging the length of two lines) whilst a task-irrelevant cue was presented within the visual display. Others have developed more “real-world” tasks such as counting ball passes or being misdirected by a magician (Hyman Jr et al., 2010; Kuhn & Tatler, 2005; Most & Astur, 2007; Neisser & Becklen, 1975; Simons & Chabris, 1999). Interactive digital games provide a useful environment for the study of attention as they are intended to guide people’s attention towards the game, and stop players from becoming distracted. Game tasks are more similar to real-world situations in that players are given a goal but they are in control over how they complete that goal. Such games last much longer than a typical IB experiment, which provides a unique opportunity to study the impact of sustained attentional engagement on the processing of task-irrelevant information.

Existing research on video games confirms that they can create sustained attentional selection but very few consider task-irrelevant processing. There is extensive work on how game playing impacts attentional performance (e.g., Boot et al., 2008; Green & Bavelier, 2006; Hubert-Wallander et al., 2011; Murphy & Spencer, 2009), which shows that games place high demands on attentional selection. Eye-tracking has been used to track players’ overt attention (El-Nasr & Yan, 2006; Sundstedt et al., 2008) to investigate their game experience and optimize graphical quality by concentrating processing resources only on attended areas (Sundstedt et al., 2004; Sundstedt et al., 2005). Just a few studies use game-like environments to investigate task-irrelevant processing. Wood and Simons (2019) used an interactive environment, similar to the video game *Frogger,* to investigate task-irrelevant processing in a spatial-inattentional blindness paradigm. Since players need to track several objects moving at different speeds, the game required their attention for several minutes. Most and Astur (2007) used a driving simulator, similar to a video game, to investigate feature-based attentional selection. However, since both these studies only presented one unexpected stimulus during the experimental time, whether the object is seen or not may be influenced by variations over time in the difficulty of the main task, which may add additional variance into the results.

In both game and non-game attentional selection studies, the task generally requires continuous processing of the stimuli. For example, in ball-bouncing tasks (Most et al., 2000; Neisser & Becklen, 1975; Simons & Chabris, 1999) participants must continuously follow the movement of the ball to count how many times it bounces. It is possible that task-irrelevant processing is at least partly dependent on participants’ time constraints and that selective attention and the consequent reduction in task-irrelevant processing is partly a pragmatic consequence of having limited attentional resource within the time available. Eitam et al. (2013) tested for relevance-based selection under minimal load, but their stimuli were only presented for 500 ms, which may also have limited the resources available. However, in the experimental context of digital games, the stimuli can play out partially or wholly in response to player actions with no need for continuous processing. In particular, in so-called *self-paced* games (Jennett et al., 2008) such as *Candy Crush Saga*, *Two Dots*, and *Civilization* players have as long as they want to make their moves and have no requirement for continuous processing or quick reaction speed. These games can still be very engaging (Dredge, 2014) and it is possible that they hold attention consistently and so present the opportunity to examine task-irrelevant processing without the need for intensive continuous processing of visual stimuli.

One problem of using digital games is that they are often complex multi-faceted systems that provide a range of player experiences. Not all games are equally engaging and players do not automatically commit their attention to them (Cutting & Cairns, 2020). Some approaches to measuring the experience of playing games (e.g., Chen, 2007) are based on the idea that being engaged in games induces a state of *Flow* (Csikszentmihalyi, 1991, 2013). During Flow the level of challenge meets the level of performance and this has been measured using experience sampling measures. However, Flow is not an accurate representation of game experience as most games involve periods of frustration and failure where challenge exceeds performance, which are then followed by easier periods (Juul, 2013; Schell, 2008). Attempts to use experience sampling with games have found that the act of sampling can interrupt the player and change the experience it was trying to measure (Kaye et al., 2018). Many validated post-game questionnaires have therefore been developed to measure different aspects of engagement, most notably the Game Engagement Questionnaire (GEQ) (Brockmyer et al., 2009), the Player Experience of Needs Satisfaction questionnaire (PENS) (Ryan et al., 2006) and the Immersion Experience Questionnaire (IEQ) (Jennett et al., 2008). These questionnaires are all widely used and, as would be hoped, show significant agreement (Denisova et al., 2016). Immersion is an aspect of engagement and has been defined as the sense of being highly engrossed in a mediated experience across multiple dimensions (Rigby et al., 2019). Brown and Cairns (2004) interviewed game players and found a common experience known as *immersion,* described by players as “When you stop thinking about the fact that you’re playing a computer game and you’re just in a computer.” Jennett et al. (2008) operationalized this by creating a validated *Immersion Experience Questionnaire (IEQ)* to measure self-reported feelings of immersion.

Jennett (2010) suggested that immersion is a form of directed attention that should moderate task-irrelevant processing. The effect of task-relevance on recall was investigated as far back as the 1930s by Zinchenko (as described by Meshcheryakov, 2008), who found that after a dual stimulus task in which one stimulus related to the activity being performed, memory was increased for the elements related to the activity. In particular, they found that “heightened interest” in the activity distracted participants from the contents of the stimulus. Jennett (2010) used a similar approach and found that task-irrelevant processing is reduced by increases in self-reported immersion across a variety of digital games. Her games were all action games that required rapid processing and fast responses. Cutting and Cairns (2020) examined task-irrelevant processing in self-paced games using the *Distractor Recognition Paradigm* (DRP). This works by surrounding the game with constantly changing irrelevant images and, after playing, players are tested on their recall of these images. In agreement with Jennett (2010), they found that task-irrelevant processing decreases with immersion.

We aimed to investigate whether sustained immersion in a self-paced digital game prevents people from processing task-irrelevant information, even when the information is presented in full view. The first two experiments examined whether game immersion modulated the processing of task-irrelevant information. The third and fourth experiments investigated how changes in the game task (i.e., the task-relevant feature) affected the type of task-irrelevant information that was being processed. All experiments in this paper conformed to the ethics procedures maintained by the Computer Science Department, University of York, UK.

## Experiments 1 and 2: Task-irrelevant processing in two games with different levels of immersion

Experiments 1 and 2 aimed to investigate the impact that actively playing a self-paced game has on the memory of task-irrelevant distractors. We also aimed to investigate how immersion affects people’s memory for task-irrelevant distractors. Jennett (2010) suggests that immersion in games is a form of selective attention, and we therefore directly examined whether different levels of immersion affect the retention of task-irrelevant stimuli. If immersion is a form of attention, then it is likely that more immersive games will direct attention more consistently and lead to reduced processing of task-irrelevant stimuli.

Participants played one of two games with different levels of immersion. We used the DRP (Cutting & Cairns, 2020) to measure different levels of task-irrelevant retention as a function of immersion. We developed two games that involved similar visual displays, but different levels of immersion. We predicted that participants will recognize more distractor images after the low-immersion game compared with the high-immersion game. Experiment 2 was a large-scale online replication of Experiment 1 to address issues with power and ecological validity. In Experiment 1 the game was played in a lab situation with participants constrained by a chin rest. Experiment 2 was delivered via a web browser on participants’ own computers. Experiment 2 was pre-registered here: https://osf.io/ew7jg.

### Method

#### Participants

In Experiment 1, 36 staff and students from the University of York with a wide range of previous game experience took part in the study. Seventeen were male and ages ranged from 18 to 57 years (*M* = 21.4). Participants were paid £6. For Experiment 2, an online pilot (n = 38) gave an effect size (d) of 0.75, which would require 132 participants to produce a power of 0.96. To allow for error, we set a target sample size of at least 160 valid participants. Our stopping rule was to collect 180 participants and discard all invalid responses; if either condition had less than 80 valid responses, we would then recruit participants one at a time until we had at least 80 in each condition. We recruited 186 participants via the online experiment platform *Prolific* on 4 May 2020*.* We rejected 26 participants – seven due to technical issues with the experiment, four due to color-blindness, 14 due to failing an attention check and one for failing a questionnaire check. This resulted in 160 participants with 80 in each condition. Of these, 77 were male, 81 female and two non-binary with ages ranging from 18–40 years (*M* = 25.8). These participants were paid £1.50. In both experiments, participants were randomly allocated to one of the two conditions. Additional demographic breakdown is available in the Online Supplementary Materials.

#### Materials

Both experiments used two different games, one with higher immersion and one with lower immersion. Apart from the difference in immersion, the games were designed to involve similar visual stimuli and similar motor actions. This experiment made use of two variants of the mobile puzzle game *Two Dots*. This is a simple self-paced puzzle game that is engaging and can be learnt quickly (Crook, 2014; Fine, 2015). The game is played on a grid of different-colored dots and the aim is to join adjacent dots of the same color and meet targets within a set number of moves.

**Fig. 1:**
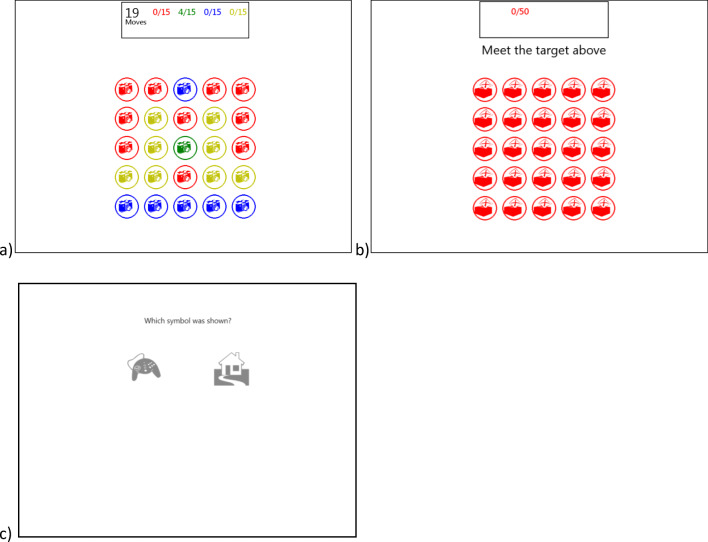
(**a**) The *High immersion game* with in-game distractors. Players have to join dots of the same color. The images inside the dots change every 5s. (**b**) The *Low immersion game* with in-game distractors has dots all the same color, which is less engaging. (c) The distractor recognition test. Participants need to choose one image from the two. One of these images has been shown to the participant during the experiment. The other has not been shown before

Two different custom variants of *Two Dots* were built and designed to be played on a desktop computer with a mouse*.* The *High immersion* game variant was a direct clone of *Two Dots* (shown in Fig. 1a). We designed a second game variant with a reduced level of immersion but similar stimulus and motor actions. To do so, we changed the *High immersion* game so that all the dots were the same color. By making all the dots identical, we made the game less engaging, which should reduce immersion even though participants are still performing the same activity of joining dots to meet a target and moving on to the next level. This game was known as the *Low immersion* game and is shown in Fig. 1b. In both of these games the dots all contained images from the *Webdings* typeface, which were irrelevant to the gameplay and changed to a different image every 5s. The images were chosen randomly for each participant from a pool of 90. Each image was shown for 5 s (as in Standing, 1973) in a unique random order.

After playing the game for 5 minutes, participants were tested on how many images they recognized. The recognition test consisted of presenting participants with 30 image pairs, one of which had been previously presented and a new one, and they were required to identify the previously presented image (Fig. 1c).

The materials in Experiment 2 were almost identical to those in Experiment 1 except the software was recoded from Python to Javascript so it would run in a web browser. Initial pilot tests suggested that participants reported higher levels of immersion than in the lab experiment, so we made the *Low immersion* game even less immersive by removing the running total of dots joined. Pilot tests also suggested that online participants may be less likely to understand how to play the *High immersion* game so we added an additional training level. We were concerned that online participants would not be motivated to get their best score in the image-recognition test so we added feedback to indicate whether their answer was correct or not. A version of the experiment that does not save data and allows choice of condition can be viewed here: http://www.joecutting.com/demos/varyImmersion/index.html

#### Design and procedure

Both experiments were a between-participants design with two conditions. A within-participants design was not suitable as participants played a puzzle game during the experiment and playing a second time would be subject to a large practice effect. The independent variable was the game each participant played*.* The main dependent variable was the number of distractors that participants recognized after the activity. Another secondary dependent variable was the Immersion Experience Questionnaire (IEQ) score for each participant’s experience of the activity.

All participants began by completing a consent form. Each participant played either the *High immersion game* or the *Low immersion* game, and participants were randomly allocated to one of the two groups. All participants started with a short tutorial. After 5 min of play the game stopped automatically, after which participants completed the on-screen distractor recognition test followed by the IEQ. Experiment 1 was displayed on a 24-in. monitor with screen dimensions of 51.5 x 32.5 cm. During the experiment participants kept their chin in a chin-rest, which was positioned 95 cm from the screen. This meant that the screen display filled 31.5° of the participant’s field of view. In Experiment 1 the IEQ was presented on an iPad away from the main game computer.

The design and procedure of Experiment 2 was identical to the lab version, except that all aspects of the experiment were performed online via participants’ web browsers. For the online experiments, participants were required to use a desktop or laptop computer rather than a phone or tablet.

### Results and discussion

**Fig. 2:**
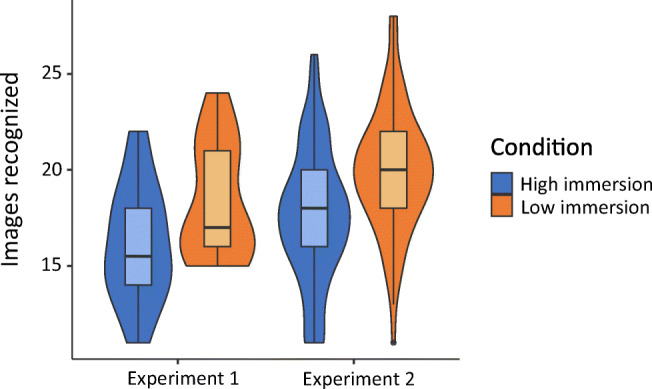
Violin plot of images recognized in Experiments 1 and 2

**Fig. 3:**
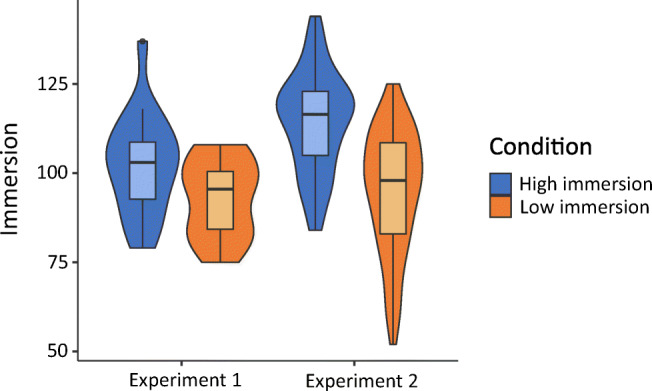
Violin plot of immersion in Experiments 1 and 2

The results of both experiments are shown in Table 1 and plotted in Figs. 2 and 3. In both experiments participants recognized significantly more images after the *Low immersion* game than the *High immersion* game (Exp. 1 t(34) = 2.22, p=.034, d = 0.74; Exp. 2 t(158) = 4.48, p<.001, d = 0.71). Immersion was also significantly higher in the *High immersion* game in both experiments (Exp. 1 t(34) = 2.28, p= .029, d = 0.76, Exp. 2 t(158) = 7.85, p<.001, d = 1.24).

**Table 1:** Results from Experiments 1 and 2

	Experiment 1 (lab)		Experiment 2 (online)	
	High immersion	Low immersion	High immersion	Low immersion
n	18	18	80	80
Images recognized				
Mean	16.1	18.3	17.6	20.0
SD	3.01	3.01	3.46	3.17
Immersion				
Mean	103	92.9	114	95.0
SD	14.2	10.8	14.0	17.0

A regression analysis on Experiment 1 showed a significant correlation between immersion and the number of images recognized; however, there was no significant correlation in Experiment 2. This analysis is included in the Online Supplementary Materials.

### Discussion

Both Experiment 1 and Experiment 2 show a clear reduction in retention of task-irrelevant images in the *High immersion* game condition, compared to the *Low immersion* condition. In the *High immersion* game participants had to match dots of the same color and the images displayed on the dots were not relevant to playing the game. To play the game, participants have to look at the images and despite spending 5 minutes looking directly at the images, they recognized very few of them in the subsequent recognition test. We previously conducted a pilot study in which participants were shown the same sequence of images but without the game element. This pilot replicated Standing's (1973) finding of extremely high recognition performance (> 90%). The low number of images recognized in the *High immersion* game shows a low level of retention when engaged in an immersive game. Indeed, retention of the images was significantly better in the *Low immersion* game. It is likely that in the *High immersion* game participants were paying attention only to the features of the dots that were needed for the game task (i.e., the colors) rather than the images. As they were not paying attention to the images, they had a low level of recall of them afterwards. Conversely, in the *Low immersion* game participants did not need to pay attention to the colors of the dots and their attention was more likely to drift onto the images so they recognized more images after the game.

In both experiments, participants were significantly more immersed in the *High immersion* game than the *Low immersion* game. The immersion questionnaire (Jennett et al., 2008) includes questions on both top-down motivations to perform the task (e.g., “How much would you say you enjoyed playing the game?”) and also the experience of lower level feelings of attention (e.g., “To what extent did you notice events taking place around you?”). As with magic tricks (Kuhn et al., 2016), it is likely that there are both top-down and bottom-up attentional mechanisms affecting the retention of task-irrelevant features and the immersion questionnaire may be capturing the effect of both of these.

There were some differences between the results of the lab-based experiment and the online replication. Participants who played the game online in Experiment 2 reported higher immersion than those who played the same game in the lab. For the *High immersion* game this difference was considerable. The online *Low immersion* game had been modified to make it even less immersive, but despite this the online *Low immersion* game had a higher mean immersion score than the lab version. In Experiment 1, a regression model found a significant main effect of immersion on the number of distractors recognized, but the same analysis in Experiment 2 found no significant effect of immersion. The online experiment had four times as many participants as the lab experiment, so these differences may be due to the larger sample size measuring the effect more precisely. However, participants reported higher levels of immersion in the online games (Exp. 1 and Exp. 2) than the lab-based studies, which may have resulted from generally higher levels of immersion when playing online.

It is likely that participants’ attention was affected by both top-down and bottom-up processes. When considering bottom-up processes, load theory (Cartwright-Finch & Lavie, 2007; Lavie, 2005; Lavie et al., 2004) predicts that, as the *High immersion* game requires higher perceptual load, participants would be less likely to be distracted, which is indeed the case. Load theory differentiates between *perceptual* load and *cognitive* load. It predicts that higher cognitive load, which may be needed for the additional puzzle elements in the *Higher immersion* game, would lead to greater distraction in the *High immersion* game, which was not found here. Participants may have overcome being distracted due to other top-down factors such as player motivation, or it is possible that the *High immersion* requires no significant cognitive load despite being a “puzzle” game. Previous studies on feature-based attention (Most & Astur, 2007; Simons & Chabris, 1999; Wood & Simons, 2019) have found higher levels of inattentional blindness when the task-irrelevant features are dissimilar to the task-relevant features. In the *High immersion* game, participants need to attend to the dot colors but not the images within them. The colors and images are distinct features and the finding that participants pay attention to one and not the other, which then affects subsequent recall, is consistent with previous feature-based attention studies. In the lower immersion game, participants do not need to pay attention to particular features so their recall of task-irrelevant features is higher.

The games played in this experiment had different levels of immersion but also had different gameplay, with the *High immersion* game offering higher difficulty than the *Low immersion* game. It is possible that higher difficulty may be partly responsible for the reduction in image recognition, as the higher difficulty may have increased cognitive load, which then reduced memory capacity (Baddeley & Hitch, 1974) and image recognition rates. However, large differences in cognitive load are unlikely as load theory (Cartwright-Finch & Lavie, 2007; Lavie, 2005; Lavie et al., 2004) would predict that higher cognitive load would lead to greater distraction, which was not found in these experiments or similar experiments by Cutting and Cairns (2020). Even so, manipulating attentional selection by changing immersion risks changes the difficulty and load demands of the task. To avoid this, the next two experiments manipulated attentional selection by changing the gameplay goal rather than the level of immersion. This allowed us to keep the difficulty and load requirements constant between conditions and remove the possibility that differences in load are partly responsible for differences in recall.

## Experiments 3 and 4: Task-irrelevant processing in two games with the same level of immersion

The next two experiments aimed to investigate the difference between task-relevant and task-irrelevant processing on image recognition, whilst controlling for immersion and processing load. To achieve this goal, participants played one of two different games that both had similar mechanics and visual stimuli, but different play goals. Both games contained identical images, but in only one of the games were the images relevant to the game task. We predicted that people should be more likely to remember images when the images were a central feature of the game, despite the games being equally immersive. Experiment 4 was a large-scale online replication of Experiment 3 to address the same issues with ecological validity that motivated Experiment 2. Experiment 4 was pre-registered here: https://osf.io/m9ycu

### Method

#### Participants

In Experiment 3, 40 students and staff from the University of York with a wide range of previous game experience took part in the study. Twenty-nine were male and 11 were women. Ages ranged from 18 to 25 years (*M* = 20.6). Participants received £6 in compensation. In Experiment 4, the target sample size was set at 160 to match Experiment 2. We used the same sampling plan and stopping rule as Experiment 2 and recruited 184 participants via the online experiment platform *Prolific* on 11 May 2020*.* We rejected 24 participants: 13 due to technical issues with the experiment, one due to color-blindness, six due to failing an attention check, two for failing a questionnaire check and two for not achieving a high enough level in the game. This resulted in 160 participants with 80 in each condition. Of these 71 were male and 89 were female, with ages ranging from 18 to 40 years (*M* = 28.0). Participants received £1.50 in compensation. In both experiments, participants were randomly allocated to one of the two conditions. Additional demographic breakdown is available in the Online Supplementary Materials.

#### Materials

**Fig. 4:**
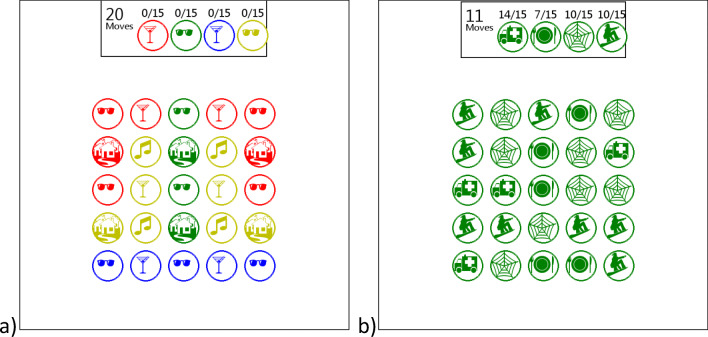
(**a**) Match colors variant of *Two Dots* in which participants join dots of the same color. (**b**) Match images variant of *Two Dots* in which participants join dots of the same image. Every 5s all the images change to a different color

As with Experiments 1 and 2, both games were variants of the game *Two Dots*. In one variant, participants join dots that are the same color and ignore the images. This variant is known as *Match colors,* and it was similar to the *High immersion* game used in the previous experiments. The only difference was that the *Match colors* game displayed four different images at the same time whereas the *High immersion* game displayed the same image in each dot. In the other variant players join dots that have the same image and ignore the colors. This variant is known as *Match images.* Both variants display four different images at the same time. Every 5 s one image changes; this happens in turn so that each image is displayed for 20 s in total. In the variant where players match the images, all of the images change color every 5 s. This is to ensure that participants are shown images in every color to make the overall stimulus as close as possible to the other game variant. We used the same recognition task as in the previous experiment. Both game variants are shown in Fig. 4.

In Experiment 4 the materials were almost identical to those in Experiment 3 except the software was recoded to run in a web browser. Pilot tests also suggested that online participants may be less likely to understand how to play the game so we added an additional training level. We were concerned that online participants would not be motivated to get their best score in the image-recognition test so we added feedback to indicate whether their answer was correct or not. A version of the experiment that does not save data and allows choice of condition can be viewed here: http://www.joecutting.com/demos/sameImmersion/index.html

#### Design and procedure

The design and procedure for these experiments were identical to those in Experiments 1 and 2, except that participants either played the *Match colors* or the *Match images* game and their performance at the game was recorded.

### Results and discussion

**Fig. 5:**
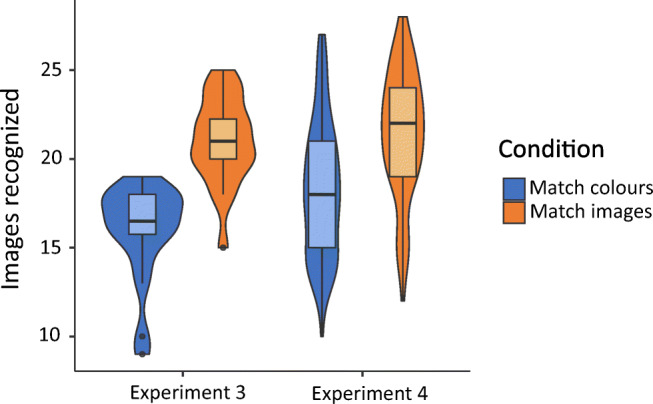
Violin plot of images recognized in Experiments 3 and 4

**Fig. 6:**
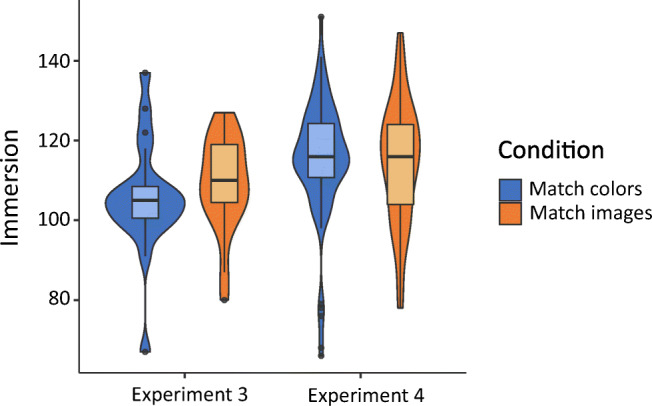
Violin plot of immersion in Experiments 3 and 4

The results of both experiments are shown in Table 2 and plotted in Figs. 5 and 6. In both experiments participants recognized significantly more images after the *Match images* game than the *Match colors* game [Exp. 3, t(38) = 6.24. p<.001, d = 1.97, Exp. 4, t(158) = 5.56, p<.001, d = 0.88] . There were no significant differences in immersion between the two different games [Exp. 3, t(38) = 0.858, p = .40, d = 0.27; Exp. 4, t(158) = -0.42, p=.68, d = 0.07], or game performance [Exp. 3, t(38)=-0.92, p=.36, d = 0.29; Exp. 4, t(158)= 0.80, p=.43, d = 0.13].

**Table 2:** Results from Experiments 3 and 4

	Experiment 3 (lab)		Experiment 4 (online)	
	Match images	Match colors	Match images	Match colors
n	18	18	80	80
Images recognized				
Mean	21.0	16.0	21.4	18.2
SD	2.45	2.67	3.54	3.75
Immersion				
Mean	109	106	115	114
SD	11.8	14.3	14.8	15.0
Game performance^a^				
Mean	8.30	7.95	8.78	8.60
SD	1.13	1.28	1.33	1.45

^a^Experiment 4 had an additional short training level that was not in Experiment 3, which means that performance is not directly comparable between the experiments

#### Regression Analysis

To investigate whether image recognition had been moderated by immersion or game performance we performed a hierarchical multiple linear regression^1^ using recommendations from Field (2013). The initial model was based on the most likely largest factor (in this case the game condition). This analysis compared three different regression models. The first model consisted of just the game condition, the second added the immersion score, and the third added the game performance. This is shown in Tables 3 and 4, and shows that the game condition is by far the strongest factor affecting distractor recognition. Neither the level of game performance or immersion score have a significant effect on the number of distractors recognized. The proportion of additional variance over the game condition due to both of these factors is also extremely low.

**Table 3:** Experiment 3: Hierarchical linear regression which shows the effect of adding different factors to a model to predict the number of distractor images recognized

Model	R	R^2^	R^2^ change	F change	Df	Significance F change
Game condition	0.711	0.506	0.506	38.927	38	<.001
Game condition and immersion	0.711	0.506	<0.01	0.001	37	.972
Game condition, immersion, performance	0.715	0.512	<0.01	0.409	36	.527

**Table 4:** Experiment 4: Hierarchical linear regression which shows the effect of adding different factors to a model to predict the number of distractor images recognized

Model	R	R^2^	R^2^ change	F change	Df	Significance F change
Game condition	.404	.163	.163	30.874	158	<.001
Game condition and immersion	.421	.177	.013	2.556	157	.112
Game condition, immersion, performance	.431	.186	.009	1.751	156	.188

1 With VIF statistics in the range 1.00–1.23 and tolerance statistics in the range 0.89–1.00

### Discussion

We aimed to investigate whether participants could recall game features when highly immersed in a game, and how game-relevant features affect the recall of task irrelevant items. In Experiments 1 and 2 it is possible that differences in the cognitive or perceptual load required by the games affected recognition rates. Here participants played games with identical game mechanics and level design, which should result in similar levels of cognitive and perceptual load. Despite this, in both Experiment 3 and Experiment 4 participants in the *Match images* condition recognized significantly more images than in the *Match colors* condition. These results illustrate that recognition is related to the relevance of the stimulus feature.

In Experiments 1 and 2, the *High immersion* game had a lower rate of distractor recognition. Baddeley and Hitch (1974) found that high cognitive load reduces memory capacity and it is possible that this game required higher cognitive load which reduces recognition memory. In Experiments 3 and 4 there were no significant differences in immersion or game performance between conditions with very small effect sizes. As the gameplay, immersion, and performance in both games were very similar, it seems unlikely that differences in cognitive load explain the difference in recognition performance reported in the different conditions. Further evidence was provided by a regression analysis that showed that differences in immersion or performance had a negligible effect on the number of distractor images recognized. As the key difference between games was the task goal, the most likely reason for the difference in recognition is the relevance of the features to the task currently being performed.

There were some differences between the results of the lab-based experiment and the online replication. As in Experiments 1 and 2 online participants consistently reported higher levels of immersion than those in the lab, participants who played the *Match Colors* game online in Experiment 4 recognized more images than those who played in the lab. These differences may be due larger sample sizes creating more robust results, but it may also be due to differences in the experimental environment. In particular, the lab-based games were played with a mouse but many online participants used a trackpad, which may have been more awkward and may have disrupted attentional selection.

Load theory (Cartwright-Finch & Lavie, 2007; Lavie, 2005; Lavie et al., 2004) predicts that since players in both games are under similar levels of cognitive and perceptual load, their levels of distraction should be similar. This is consistent with our findings as it is likely that participants have their attention held by the particular game task and are not distracted by other features present in the game. In the *Match colors* game this means they only pay attention to the colors and are not distracted by the images in the game, and so do not recognize them afterwards. These findings are also consistent with previous feature-based attention studies (e.g., Most & Astur, 2007; Simons & Chabris, 1999), which show that participants attend only to an “attentional set” of task-relevant features. At the beginning of the game participants would create the attentional set required for the particular game task and only pay attention to those features within that set and so not recall features outside the set.

## General discussion and conclusions

We aimed to investigate whether playing a self-paced digital game could create sustained attentional selection that would prevent people from processing task-irrelevant features, which are presented in full view. Our first two experiments looked at the processing of task-irrelevant information in games with different levels of immersion. The third and fourth experiments investigated how changes in the game task (i.e., the task-relevant feature) without changes in immersion affected the type of information that could be retained after the task.

The experiments used the distractor recognition paradigm to show that even a simple, self-paced game like *Two Dots* can direct players’ attention for a sustained period of time. During both the *High immersion* and *Match colors* games there is sustained attentional selection away from task-irrelevant features for the whole game, such that visible features not needed for the task are not recalled. This differs from existing task-irrelevant processing paradigms that present the task-irrelevant information for a few seconds and distract participants with tasks that require continuous processing within a short period of time. For example, in Most et al.'s (2000) “Sustained inattentional blindness” paradigm, trials last only 15s, and the unexpected shape is visible for only 5s. Similarly, in Simons and Chabris' (1999) well known “Gorillas in our midst” study, the trial lasted only 75s, with the unexpected gorilla visible for only 5s, and participants are required to perform an intensive continuous processing task (i.e., counting ball bounces).

In contrast, our experiments employ the DRP to show that participants’ attention is diverted from task-irrelevant features for the full 5 minutes of the experiment, and since the game is completely self-paced, the task has no requirement for continuous fast processing. It is likely that participants form an “attentional set” (Most, 2010) of features that they should attend to and disregard other features. This may be similar to the process of misdirection in magic tricks (Kuhn et al., 2008), in which the magician creates a set of expectations so people’s attentional set does not contain the important features of the trick. The DRP also allows more sensitive quantification of attentional selection over time for each participant than previous IB paradigms. The number of distractors recognized quantifies how consistently attention is diverted away from those distractors over the time of the experiment. In contrast, existing IB paradigms (e.g., Most & Astur, 2007; Most et al., 2000; Simons & Chabris, 1999; Wood & Simons, 2019) only record whether the participant noticed the unexpected stimulus or not, which can only be quantified by considering the percentage of participants who notice the stimulus.

The experiments required participants to play a game that had a central goal (“get to the highest level”), but participants had control over how they achieved this goal. Many previous IB studies (e.g., Most et al., 2000; Simons & Chabris, 1999) use more “closed” tasks in which participants have little control over how the task is completed. Some studies (e.g., Most & Astur, 2007; Wood & Simons, 2019) give participants a small amount of agency in how they complete the task, but these are very low-level decisions and the majority of the task is continuous information processing in reaction to a stimulus. In the experiments reported here, participants had a high degree of autonomy about how to complete the task as they could make choices about which dots to join, and they did this at a pace of their own choosing. The first two experiments showed that in situations where participants have a high degree of task autonomy, self-reported feelings of immersion in the task are a key factor in the recall of task-irrelevant features. Immersion is an aspect of engagement that corresponds to self-reported feelings of being engrossed in the game. Future studies that investigate attention in situations where participants have a high degree of autonomy may need to consider how engaged participants are in the task, and take steps to ensure that they do not become disengaged.

Experiments 3 and 4 show that participants remember features even when engaged in the game, as long as those features are relevant to the central task. In the *Match colors* game, the images are not needed for the task and are not attended so are not recognized afterwards. The number of images recognized in the *Match colors* game was low, which indicates sustained attentional selection away from the image feature of the dots for the whole 5 minutes of game play. Both games had the same game play and there were no significant differences in performance or immersion between conditions. This suggests that differences in the recall of distractors were not due to differences in cognitive or perceptual load. This is similar to Eitam et al.'s (2013) finding that artificial grammar learning occurred only for task-relevant features regardless of spatial attention or the availability of attentional resources. We suggest that the differences between games were due to the differences in the “attentional set” (Most, 2010; Most & Astur, 2007). Most’s attentional set experiments were conducted in fast-moving environments in which the task required continuous processing. It is possible that in those situations the attentional set is partly a pragmatic response to a shortage of processing resources. In the second experiment both games were self-paced so participants were under no time pressure and they could play at their desired speed. It is possible that once the task requirements were clear, participants created a minimal “efficient attentional set” for the game that they were playing, despite having the processing resources available to pay attention to a wider range of features. This minimal attentional set then led to the reduced recall of images after the game.

Our paradigm differs from the classical IB paradigm in that our participants were fully aware of the presence of the irrelevant stimuli, but they disregarded them. In the classical IB paradigm participants do not know beforehand that the irrelevant stimulus will appear. Since our participants know that the images are there but suppress them, it could be argued that our paradigm results in *attentional suppression* rather than inattentional blindness. Liu (2019) describes how attention to a particular stimulus feature (such as its color) can suppress processing of surrounding non-attended features. This could be the same process taking part in the second experiment. Chelazzi et al. (2019) differentiate between three different states of attention – attended, not attended, and a third state where attention is suppressed. They conclude that attentional suppression uses different neuronal mechanisms from non-attending as during suppression the attentional set may contain information about the stimuli to be suppressed as well as the stimuli to be attended to (Arita et al., 2012). Most attentional suppression research tends to use a split-second reaction-time paradigm, but attentional suppression has also been studied in an inattentional blindness paradigm (Wood & Simons, 2017). In Experiments 3 and 4 participants were able to direct their attention away from the images without any performance penalty in the game, which may be because their attentional set also suppressed attention to those images. This would imply that participants in these experiments add the images to their “suppression attentional set,” which thus reduces the processing allocated to those images. It is possible that unattended images may have been perceived but not remembered due to *inattentional amnesia* (Wolfe, 1999). Future studies could use Butler and Klein's (2009) category association and perceptual identification tests instead of a recognition test to investigate this possibility.

Our findings have a number of implications for real-world applications. Serious games aim to educate or persuade players rather than just entertain (Anderson et al., 2010; Baranowski et al., 2008; Susi et al., 2007). One of the most successful design recommendations for effective serious games has been that the content to be communicated is *intrinsic* to the gameplay, rather than just being present on the screen at some point in the game (Deterding, 2015; Echeverría et al., 2012; Habgood & Ainsworth, 2011). Our findings support this recommendation and our results illustrate that players may only pay attention to features that are important to the gameplay. If content is not intrinsic to the gameplay, then it will result in less processing and retention. This conclusion is supported by studies into the “split attention principle” (Ayres & Sweller, 2005; Chandler & Sweller, 1992), which show that asking learners to divide their attention between different features of a learning stimulus results in reduced learning.

There are also implications for advertising within games, which is a growing source of revenue for games companies (Nelson et al., 2004). Our findings suggest that if players are fully immersed in a game then they are unlikely to pay attention to in-game adverts that are separate to the main gameplay. This lack of attention could reduce processing and retention of the advert after the game. However, if players are less immersed in the game itself then their attention is more likely to drift onto the adverts, so it may be advantageous to put in game adverts in less immersive parts of the game. It may also be that there are many other non-game self-paced tasks that, despite appearing to be of low intensity, also create an attentional set, which ensures that task-irrelevant features are not processed despite being within overt attention.

In summary, it is widely known that games hold players’ attention away from their surroundings and onto the game. These experiments show that even self-paced games create sustained attentional selection onto task-relevant game features, which then affects processing and retention of the unattended features. The mechanisms behind this process of sustained attentional selection and the implications for how players experience games are still largely unknown and a rich area for future work.
